# Unlocking Fast Na^+^ Migration in F-Doped O3-Type Cathodes via First-Principles Calculations

**DOI:** 10.3390/nano16090563

**Published:** 2026-05-02

**Authors:** Hong Wu, Yanjian Guo, Guannan Zu, Yong Li

**Affiliations:** 1 State Key Laboratory of Green Building, Xi’an University of Architecture & Technology, Xi’an 710055, China; wuhong1969@xauat.edu.cn (H.W.); gyj@xauat.edu.cn (Y.G.); gnzu21@xauat.edu.cn (G.Z.); 2 Shaanxi Key Laboratory of Nanomaterials and Nanotechnology, Xi’an University of Architecture and Technology, Xi’an 710055, China

**Keywords:** sodium ion batteries, O3-type layered oxide, elemental doping, first-principles calculations, electronic structure

## Abstract

O3-type layered transition-metal oxides are widely regarded as promising cathode materials for sodium-ion batteries due to their intrinsically high sodium content and favorable energy density. Nevertheless, their practical rate capability is hindered by sluggish Na^+^ transport and relatively high diffusion barriers. To address this issue, elemental substitution has emerged as an effective modification strategy. In this work, fluorine (F), characterized by strong electronegativity and a small ionic radius, is introduced to partially substitute oxygen in the bulk lattice of O3-type NaNi_1/3_Fe_1/3_Mn_1/3_O_2_ (NNFM). First-principles calculations demonstrate that F incorporation leads to an expansion of the interlayer spacing along the c-axis and a weakening of Na–O interactions, both of which facilitate Na^+^ migration. Among the considered configurations, Mn-adjacent substitution exhibits the lowest formation energy, indicating enhanced thermodynamic stability. Furthermore, electronic structure analysis reveals a reduced band gap (from 0.515 eV to 0.342–0.356 eV) and strengthened O-2p/Mn-3d orbital hybridization, contributing to improved electronic conductivity. These findings provide atomistic insights into F-induced modulation mechanisms and suggest an effective pathway for optimizing Na^+^ transport in O3-type cathodes.

## 1. Introduction

Sodium-ion batteries (SIBs) have attracted increasing attention as viable alternatives to lithium-ion systems, primarily due to the natural abundance, low cost, and wide availability of sodium resources [1,2,3,4]. Within these systems, cathode materials play a decisive role in determining both energy density and power output [5,6]. Layered transition-metal oxides (Na_x_TMO_2_, 0 < x ≤ 1) are particularly appealing because of their high theoretical capacity, structural tunability, and relatively straightforward synthesis routes [7,8,9].

Compared with P2-type phases, O3-type layered oxides (0.8 < x ≤ 1) typically accommodate a higher sodium content, which is advantageous for achieving large reversible capacity and high coulombic efficiency in full-cell configurations [10,11,12,13,14,15]. However, O3-type layered oxides still encounter scientific challenges associated with kinetic barriers because of slow Na^+^ migration [16,17,18,19]. In O3 frameworks, sodium ions initially occupy octahedral sites and migrate via an intermediate tetrahedral position (oct → tet → oct) [20]. This migration route causes high energy barriers because of the large Na^+^ ion size difference with the narrow tetrahedral interstitial sites [21].

In this work, we systematically investigate F-doped O3-type NaNi_1/3_Fe_1/3_Mn_1/3_O_2_ (denoted as NNFM) using first-principles DFT + U calculations. Three doping sites (Ni, Fe, and Mn) are constructed and evaluated to comprehend the impact of F alteration on structural stability, electronic properties, and Na^+^ migration kinetics. Formation energy calculations are performed to identify the most stable configuration. The evolution of lattice parameters, bond lengths, and the density of electronic states is analyzed to clarify the structural and electronic modifications induced by F doping. The energy barriers for Na^+^ migration are calculated by employing the climbing-image nudged elastic band (CI-NEB) method. Furthermore, differential charge density, Bader charge, and spin magnetic moment analyses are conducted to reveal the charge transfer behavior and redox mechanism during the desodiation process. This study provides atomic-scale insights into the role of F doping in enhancing the electrochemical performance of O3-type layered oxide cathodes and offers a simple yet effective strategy for modulating Na^+^ migration paths toward high-rate sodium-ion batteries.

## 2. Structural Models and Computational Methods

All density functional theory (DFT) calculations were conducted utilizing the Vienna Ab initio Simulation Package (VASP) version 5.4.4 (VASP Software GmbH, Vienna, Austria)and Visualization for Electronic and Structural Analysis (VESTA) version 3.5.8 (National Institute for Materials Science (NIMS), Tsukuba, Japan). The projector augmented wave (PAW) approach and the generalized gradient approximation (GGA) with the Perdew—Burke—Ernzerhof (PBE) functional were employed. To properly describe the strong on-site Coulomb interactions of transition metal d electrons, a Hubbard U correction (GGA + U) was applied with U values of 6.0 eV for Ni, 4.0 eV for Fe, and 3.9 eV for Mn. A plane-wave cutoff energy of 500 eV was used, and ionic relaxations were continued until the atomic forces fell below 0.02 eV/Å. Brillouin zone integration employed a 4 × 4 × 3 k-point mesh. A 3 × 3 × 1 supercell of the O3-type NaNi_1/3_Fe_1/3_Mn_1/3_O_2_ (NNFM) phase, consisting of 27 Na, 9 Ni, 9 Fe, 9 Mn, and 54 O atoms, was constructed. F doping was introduced by replacing one O atom with one F atom near the Ni, Fe, or Mn site, giving rise to three models labelled NNFM-Ni, NNFM-Fe, and NNFM-Mn, respectively. Then, the CI-NEB method was used to calculate the Na^+^ migration barriers.

## 3. Results and Discussion

**Figure 1:**
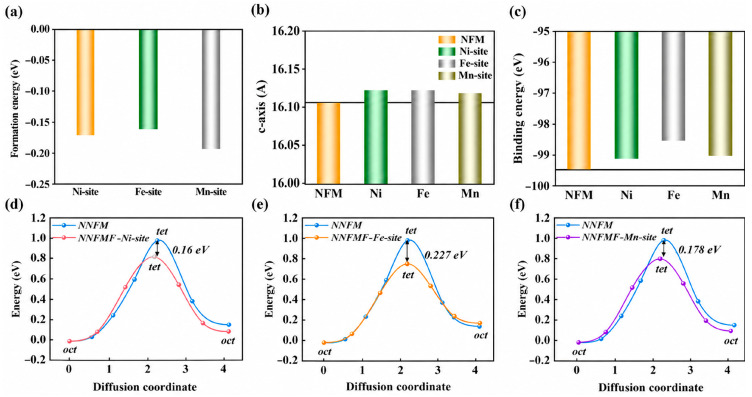
(**a**) Formation energies of different systems; (**b**) c-axis lengths of different systems; (**c**) Binding energies of different systems for sodium ions; (**d**–**f**) Comparison of the diffusion energy barriers of the pristine NNFM and the different doped systems.

To verify the accuracy of our computational approach, we first optimized the supercell structure of pristine NNFM. The resulting lattice parameters are listed in Table 1: a = 3.0221 Å, b = 3.0233 Å, c = 16.1063 Å, and V = 127.4243 Å^3^. These calculated values are consistent with the experimental data, confirming the dependability of our DFT + U settings. We constructed three doping configurations by replacing a single O atom with an F atom at positions adjacent to Ni, Fe, or Mn, referred to as NNFM-Ni, NNFM-Fe, and NNFM-Mn, respectively. Their thermodynamic stability was assessed by calculating the formation energy. As presented in Figure 1a, the formation energies for NNFM-Ni, NNFM-Fe, and NNFM-Mn are −0.169 eV, −0.162 eV, and −0.194 eV. Among these, NNFM-Mn shows the most negative value, indicating that this configuration is the most stable. Formation energy is a widely used thermodynamic stability indicator. Phonon dispersion calculations, while desirable for confirming dynamic stability, are computationally prohibitive for our 3 × 3 × 1 supercell (108 atoms) and are therefore planned for future work. Figure 1b compares the c-axis lattice parameters of the pristine and F-doped systems. Compared with pristine NNFM (16.1063 Å), the *c*-axis expands to 16.1236 Å for NNFM-Ni, 16.1232 Å for NNFM-Fe, and 16.1195 Å for NNFM-Mn, confirming that F doping effectively enlarges the interlayer spacing.

The expansion of the *c*-axis directly affects Na^+^ binding and site preference. Specifically, the enlarged interlayer distance weakens the electrostatic attraction between Na^+^ and surrounding oxygen ions, which is reflected by an elongation of the Na–O bond length (from 2.3268 Å in pristine NNFM to 2.3465 Å in NNFM-Fe). Consequently, the binding energy of Na^+^ decreases, facilitating Na^+^ extraction. Regarding site preference, it is expected that the Na_e/Na_f ratio increases with c-axis expansion, suggesting that a wider interlayer spacing favors Na occupation at edge-sharing sites.

In addition to the c-axis, the ease of Na^+^ diffusion also depends on how strongly the host framework binds to sodium ions [22]. We computed the total binding energies between the desodiated framework and all Na^+^ ions for each model, with the results displayed in Figure 1c. The values are −99.114 eV (NNFM-Ni), −98.534 eV (NNFM-Fe), and −99.025 eV (NNFM-Mn). Given that each supercell contains 27 Na atoms, the corresponding binding energy per Na atom is approximately −3.671 eV, −3.649 eV, and −3.667 eV, respectively.

The Na^+^ migration barriers were evaluated using the CI-NEB approach. As shown in Figure 1d–f, all F-doped systems exhibit significantly reduced diffusion barriers compared to pristine NNFM. The NNFM-Fe configuration exhibits the largest reduction in diffusion barrier, which is echoed by its notable c-axis expansion. It should be noted that the oct → tet → oct pathway represents the most commonly reported Na^+^ migration channel in layered oxide cathodes. Exploring alternative or three-dimensional diffusion networks at different charge states remains an important direction for future work.

**Figure 2:**
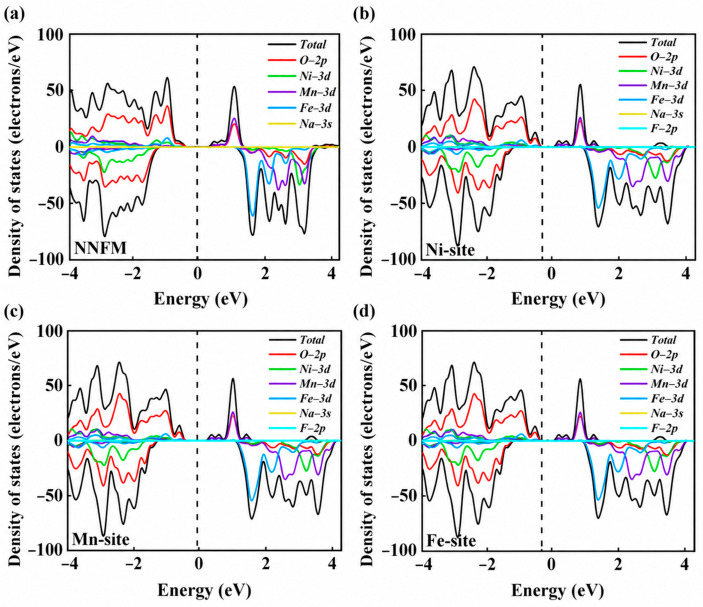
The total density of states (TDOS) and partial density of states (PDOS) of (**a**) NNFM(NaNi_1/3_Fe_1/3_Mn_1/3_O_2_) and different doped systems (**b**) NNFM-Ni, (**c**) NNFM-Mn, and (**d**) NNFM-Fe. The Fermi level is set at zero and indicated by a dashed line.

To evaluate the electronic properties of the pristine and F-doped materials, we calculated their density of states (DOS). As shown in Figure 2a, density-of-states calculations reveal that pristine NNFM exhibits a band gap of 0.515 eV, indicative of semiconducting behavior [23]. After F doping, the band gap narrows significantly: 0.343 eV for NNFM-Ni, 0.356 eV for NNFM-Mn, and 0.342 eV for NNFM-Fe (Figure 2b–d). The narrowed band gap suggests enhanced accessibility of electronic states near the Fermi level, which promotes charge transport [24,25]. The valence-band region is primarily dominated by O-2p states, whereas Mn-3d states contribute significantly to the conduction band, implying that charge transfer mainly occurs through O-2p–Mn-3d hybridization [26,27].

To further illustrate the bonding interaction induced by F doping, the differential charge density for NNFM-Fe was calculated and is presented in Figure S1. The isosurfaces in blue and yellow stand for charge reduction and increase, respectively. Charge accumulates around the F atom, while charge is depleted from the neighboring transition metal atoms (Ni, Fe, Mn). This demonstrates a strong ionic-covalent bonding interaction between F and the surrounding metals, confirming the structural stabilization role of F doping within the transition metal layer [28,29].

The structural stability can also be assessed by evaluating the migration barriers of transition metal ions (e.g., Fe) to alkali metal layer (AML) sites. Following the methodology reported by Oh et al., such calculations provide valuable insights into suppressing irreversible cation migration [30]. Although the required calculations are beyond the scope of the current study due to the large supercell size and computational cost, the strong M–O bond strengthening observed in our differential charge density analysis (Figure S1) suggests that F doping likely increases the Fe migration barrier, thereby enhancing structural stability. This will be systematically investigated in future work.

A series of NaZNi9Fe9Mn9O53F1 (Z = 27, 20, 18, 14, 7 and 0) supercells corresponding to sodium contents of x = 1.0, 0.75, 0.67, 0.50, 0.25 and 0, respectively, have been analyzed for their electronic properties through density—functional theory calculations [31]. An analysis of the total density of states depicted in Figure S2 shows a gradual decline in the peak located to the left of the Fermi energy level, along with a concurrent increase in the peak on the right side, suggesting a continuous charge compensation process during desodiation [32].

**Figure 3:**
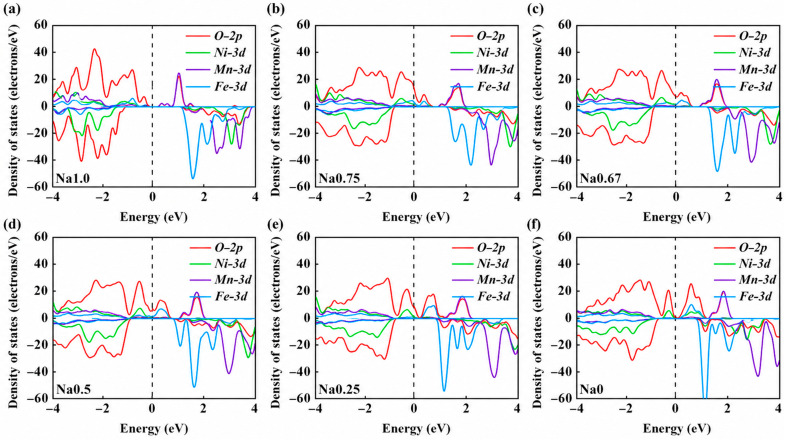
Partial density of states (PDOS) of NNFM-Fe system at different sodium contents: (**a**) x = 1.0, (**b**) 0.75, (**c**) 0.67, (**d**) 0.50, (**e**) 0.25, (**f**) 0.

Figure 3 presents the projected density of states (PDOS) of TM-3d and O-2p orbitals. During Na^+^ extraction, the Fe-related states remain consistently separated from the Fermi level in both occupied and unoccupied regions, suggesting that the Fe^3+^ oxidation state is largely preserved throughout the process [33]. In contrast, the Mn-3d and O-2p states exhibit a progressive shift from the valence band toward the conduction band, indicating their active involvement in the redox process. At lower sodium content (x < 0.25), additional contributions from O-2p states, along with partial involvement of Ni-3d orbitals, become evident. As a result, charge compensation is primarily governed by the Mn^3+/4+^ redox couple and oxygen anions over most of the charge–discharge cycle, whereas Ni^2+/3+/4+^ redox reactions are mainly activated at the final stage of charging.

The structural evolution during desodiation was investigated to assess the cycling stability. Figure S3 explores the changes in lattice parameters during the Na^+^ removal process. As illustrated in Figure S4b, the lattice constant a keeps decreasing. More critically, the variation of the lattice constant c, which directly influences Na^+^ diffusion, is shown in Figure S4c. It increases initially with Na^+^ detachment but decreases when the Na content falls below 50%. To understand this non-monotonic change, the spacing of the Na and transition metal (TM) layers was analyzed (Figure S4a,e). The initial increase in the c-axis is correlated with an expansion of the Na-layer spacing, likely due to the weakening of Na–O bonds. The subsequent contraction at low Na content is attributed to the collapse of the Na layer and a concurrent shortening of the TM–O bonds, which reduces the TM-layer spacing. Notably, the *c*-axis and layer spacings in the F-doped structure contract less severely than those in the pristine system during deep desodiation (Figure S4a,c,e). This clearly indicates that F doping effectively mitigates layer sliding and structural deformation [34]. Figure S4d shows the volume (V) variation with Na concentration, which follows a decreasing trend for both systems, consistent with the overall structural contraction.

**Figure 4:**
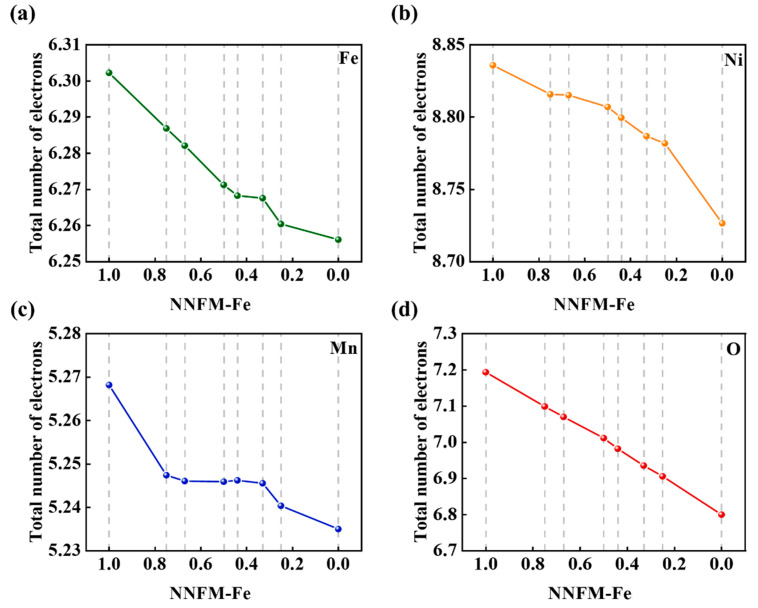
Average Bader charges of (**a**) Fe, (**b**) Ni, (**c**) Mn, and (**d**) O in NNFM-Fe at different sodium contents.

To further demonstrate the charge compensation law, the evolution of Bader charges and spin magnetic moments was analyzed. Figure 4 presents the average Bader charges of O, Mn, Fe, and Ni in the NNFM-Fe system at different sodium contents [31]. When the sodium content decreases from 1.0 to approximately 0.44, the Bader charge of Mn changes only slightly, indicating its limited involvement in charge compensation at this stage, where O and Fe serve as the primary charge donors [31]. However, when the sodium content falls below 0.44, the electron count on Mn ions decreases sharply, signifying that Mn becomes the dominant charge compensation center in this later stage. A complementary perspective is provided by the analysis of spin magnetic moments, plotted in Figure S5. The trends observed in the average magnetic moments of O, Mn, Fe, and Ni are fully consistent with the Bader charge analysis. Specifically, the magnetic moment of oxygen remains small and stable between −0.2 and 0.1 μB throughout the entire process. This confirms that oxygen maintains its O2-state, and the strong hybridization between O-2p and Mn-3d orbitals, enhanced by F doping [31], effectively stabilizes the oxygen lattice.

**Figure 5:**
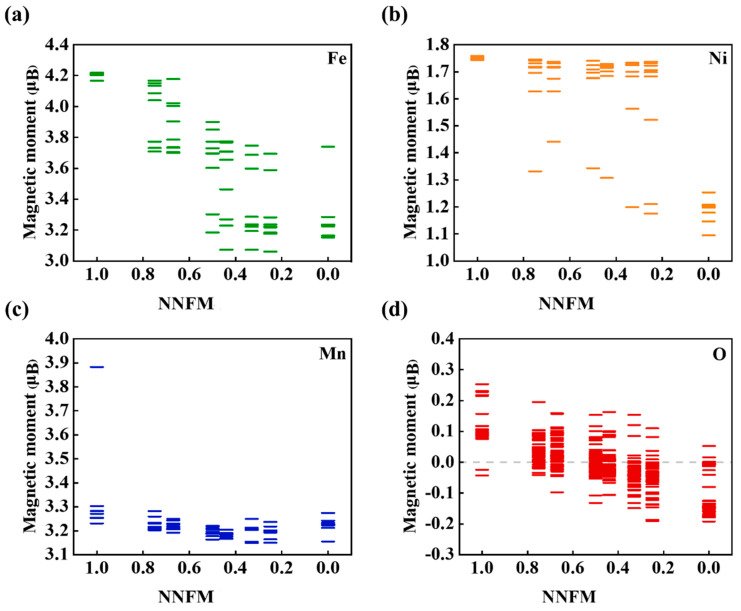
Individual atomic magnetic moments of (**a**) Fe, (**b**) Ni, (**c**) Mn, and (**d**) O in the NNFM-Fe system at different sodium contents. Red, blue, green, and orange colors represent O, Mn, Fe, and Ni atoms, respectively.

The variation of individual atomic magnetic moments offers detailed insight into valence changes, as shown in Figure 5. At a sodium (Na) content of 0.67, the magnetic moment of oxygen (O) ions remains stable within the range of −0.1 to 0.2 μB, suggesting that the majority of O exists in the −2 valence state. As the sodium content decreases, the magnetic moments of O ions become more concentrated, stabilizing within the range of −0.2 to 0.1 μB [31]. Regarding manganese (Mn) ions, the magnetic moments enable us to infer that when the sodium content is greater than 0.25, trivalent and tetravalent manganese co—exist (Mn^3+^ with ~4 μB and Mn^4+^ with ~3 μB). The initial state (Na0.67) contains only Fe^3+^ (characterized by a magnetic moment of ~5 μB). The electron arrangement of Ni^2+^ leads to a magnetic moment of ~2 μB, which is observed in the initial material. Therefore, the charge compensation during desodiation is sequentially provided by the oxidation of Fe^3+^, Mn^3+/4+^, and finally Ni^2+/3+/4+^ at deep charge, while the oxygen anions remain largely inactive.

In addition to bulk stability, surface moisture resistance is critical for practical cathode materials. Inspired by the DFT-based H_2_O adsorption analysis reported by Oh et al., we recognize that evaluating H_2_O binding energies on the surface before and after F doping would provide valuable insights into air stability [35]. However, such calculations require large surface slab models and are computationally intensive. Given the strong electronegativity of F and its tendency to polarize surrounding electron density (as shown in Figure S1), we speculate that F doping may reduce surface basicity and weaken H_2_O adsorption, similar to the effect of Nb substitution in the reference study. This remains an important direction for our future research.

## 4. Conclusions

This research utilizes first—principles calculations to investigate the formation energy, structural evolution, and Na^+^ diffusion kinetics of F-doped O3-type NaNi_1/3_Fe_1/3_Mn_1/3_O_2_. The NNFM-Fe model with optimal comprehensive performance is identified. The results show that the addition of F element reduces the formation energy and enhances the structural stability when doped at the Mn site. Through analyzing variations in lattice parameters and bond lengths, it is observed that F doping effectively expands the interlayer spacing along the c-axis and weakens the Na–O interaction, which facilitates Na^+^ detachment and migration. Moreover, the CI-NEB calculations confirm a significant reduction in the Na^+^ migration energy barrier, particularly for the NNFM-Fe configuration. Computations of the density of states reveal a reduced band gap and enhanced hybridization between O-2p and Mn-3d orbitals after doping, indicating a remarkable enhancement in the material’s electronic conductivity [31,36]. The analysis of Bader charges and magnetic moments elucidates the charge compensation mechanism during desodiation, which proceeds sequentially from Fe and Mn to Ni redox, while the oxygen lattice remains stable. These research findings are of significant importance in offering essential theoretical backing for the design and optimization of F-doped O3-type cathode materials for sodium-ion batteries.

## Figures and Tables

**Table 1:** Experimental lattice parameters and calculated lattice parameters.

Sample		NNFM	NNFM	NNFM-Ni	NNFM-Fe	NNFM-Mn
entry 1		Experimental	Calculated	Calculated	Calculated	Calculated
Phase		O3	O3	O3	O3	O3
Space Group		R-3m	R-3m	R-3m	R-3m	R-3m
lattice parameters	a (Å)	2.9635	3.0221	3.0303	3.0251	3.0316
	b (Å)	/	3.0233	3.0325	3.0321	3.0260
	c (Å)	16.1365	16.10632	16.1236	16.1232	16.1195
	α (Å)	/	90.0060	89.8917	90.1395	89.9637
	β (Å)	/	89.9938	90.1349	89.9681	89.9138
	γ (Å)	/	120.0143	120.1494	119.9417	119.9746
	V (Å)	122.733	127.4243	128.1228	128.1460	128.0936

## Data Availability

The data presented in this study are available on request from the corresponding author due to privacy or ethical restrictions.

## Supplementary Materials

The following supporting information can be downloaded at: https://www.mdpi.com/article/10.3390/nano16090563/s1, Figure S1: Three-dimensional differential charge density of NNFM-Fesite: (a) top view and (b) side; Figure S2: Total density of states (TDOS) of NNFM-Fesite at different sodium contents (x = 1.0, 0.75, 0.67, 0.50, 0.25, 0); Figure S3: Crystal structure diagram of NNFM Fe site; Changes in lattice parameters of NNFM and NNFM Fe site during the desodiation process; Figure S4: (a) transition metal (TM)-layer spacing, (b) a-axis, (c) c-axis, (d) volume V; (e) Na-layer spacing; Figure S5: Average magnetic moments of (a) Fe, (b) Ni, (c) Mn, and (d) O in NNFM-Fe site at different sodium contents.
