# Supplementary material for: Unlocking Fast Na+ Migration in F-Doped O3-Type Cathodes via First-Principles Calculations
Source: Nanomaterials (Basel). 2026 May 2;16(9):563. doi: 10.3390/nano16090563 (PMC13164706; doi:10.3390/nano16090563)
Supplement: Supplementary file 1 [file nanomaterials-16-00563-s001.zip › nanomaterials-4256020-supplementary.pdf]

Unlocking Fast Na^+ Migration in F-Doped O3-Type Cathodes via First-Principles Calculations

Hong Wu [1,2], Yanjian Guo [1,2] and Guannan Zu [1,2], Yong Li [1,2]*

1 State Key Laboratory of Green Building, Xi'an University of Architecture & Technology, Xi'an 710055, China; wuhong1969@xauat.edu.cn (H.W.); gyj@xauat.edu.cn (Y.G.); gnzu21@xauat.edu.cn (G.Z.)

2 Shaanxi Key Laboratory of Nanomaterials and Nanotechnology, Xi'an University of Architecture and Technology, Xi'an 710055, China

* Correspondence: xauatli@163.com

Computational methods

The effect of F doping on the electronic structure and crystal structure was simulated by DFT calculations with the Vienna ab initio simulation package (VASP). The projector augmented wave (PAW) method and generalized gradient approximation (GGA) with Perdew-Burke-Ernzerhof (PBE) functional and GGA+U extension were used, in which the U values applied for Ni, Fe, and Mn were 6.0, 4.0, and 3.9 eV, respectively, to correct the strong d electrons of transition metals. Structure optimization was considered complete when the plane-wave energy cutoff was 500 eV and the force convergence criterion for relaxation was 0.02 eV/Å. A 4×4×3 k-point mesh was used for Brillouin zone integration. Simulations were performed on a 3×3×1 supercell of O3-type $NaNi_{1/3}Fe_{1/3}Mn_{1/3}O_2$ (denoted as NNFM) containing 27 Na, 9 Ni, 9 Fe, 9 Mn, and 54 O atoms. F doping was modeled by substituting one O atom with one F atom at sites adjacent to Ni, Fe, or Mn, denoted as NNFM-Ni, NNFM-Fe, and NNFM-Mn, respectively. The climbing-image nudged elastic band (CI-NEB) method was employed to calculate the Na^+ diffusion energy barriers..

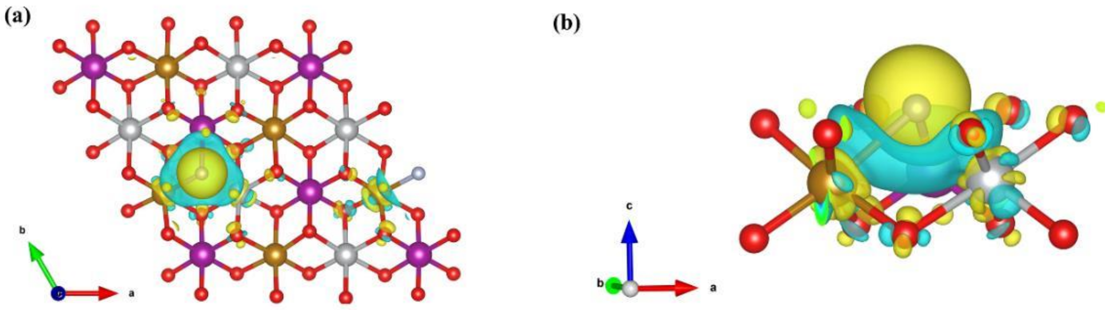

Figure S1. Three-dimensional differential charge density of NNFM-Fesite: (a) top view and (b) side view.

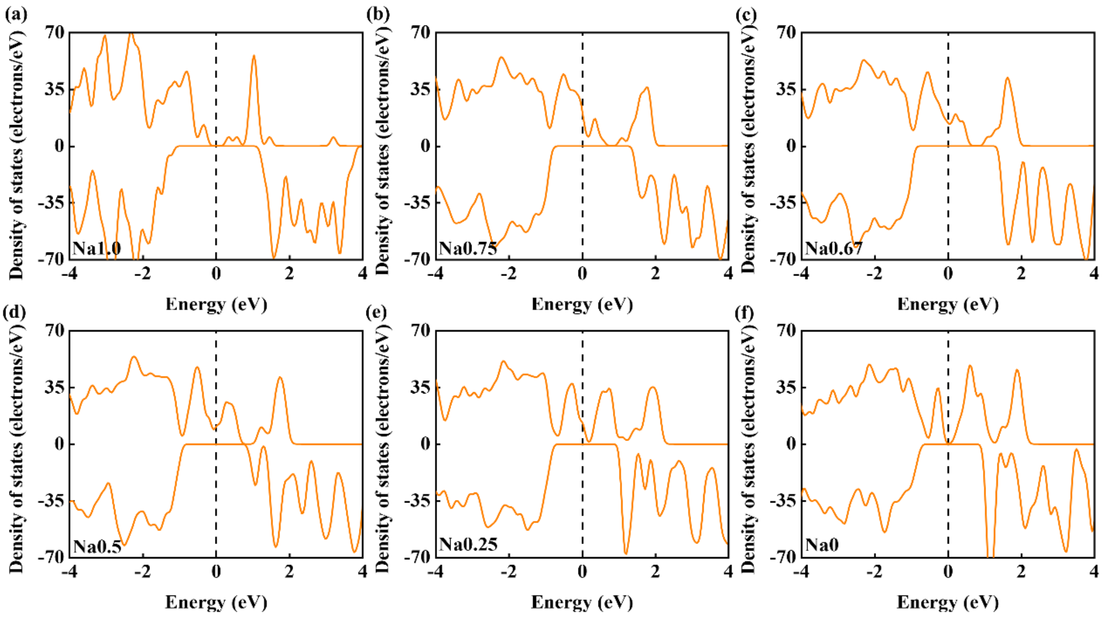

Figure S2. Total density of states (TDOS) of NNFM-$_{Fesite}$ at different sodium contents (x = 1.0, 0.75, 0.67, 0.50, 0.25, 0).

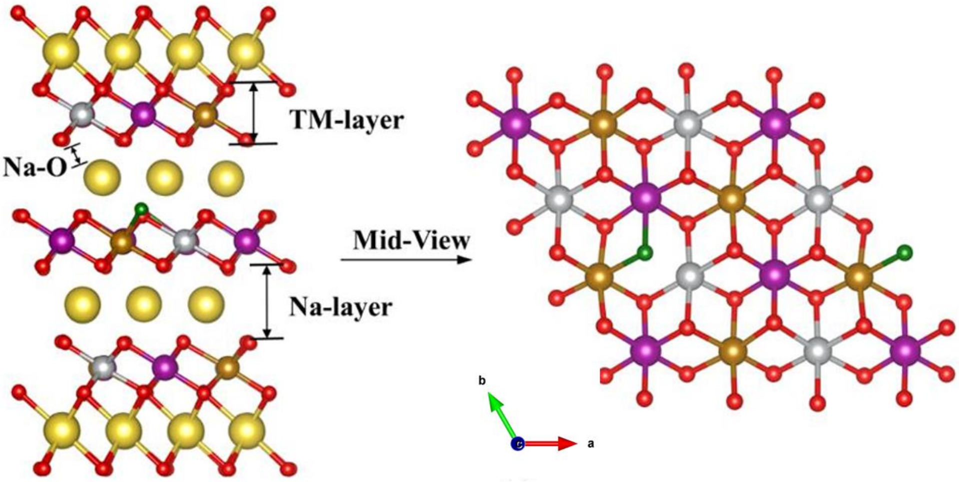

Figure S3. Crystal structure diagram of NNFM $_{Fe\ site}$; Changes in lattice parameters of NNFM and NNFM $_{Fe\ site}$ during the desodiation process

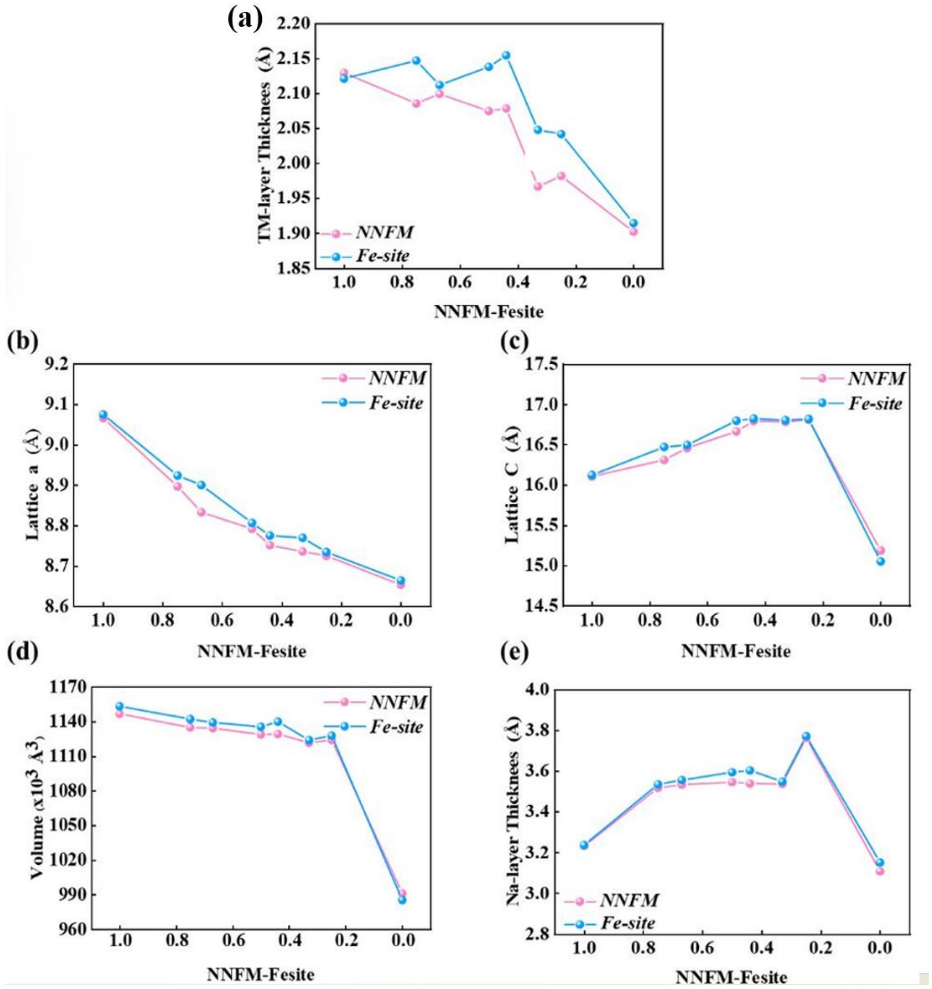

Figure S4. (a) transition metal (TM)-layer spacing, (b) a-axis, (c) c-axis, (d) volume V; (e) Na-layer spacing.

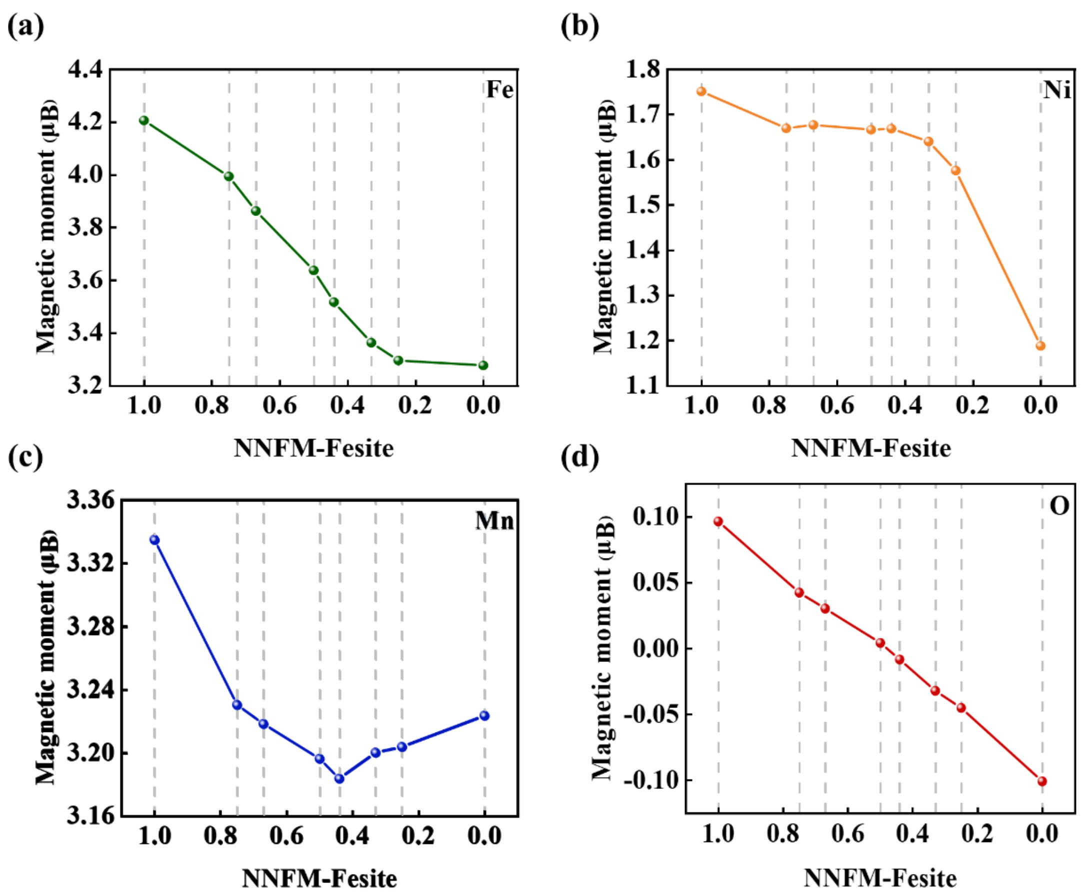

Figure **S5**. Average magnetic moments of (a) Fe, (b) Ni, (c) Mn, and (d) O in NNFM-$_{Fe\ site}$ at different sodium contents.
